# Redox State of Human Serum Albumin and Inflammatory Biomarkers in Hemodialysis Patients with Secondary Hyperparathyroidism During Oral Calcitriol Supplementation for Vitamin D

**DOI:** 10.2174/1874104501812010098

**Published:** 2018-10-18

**Authors:** Wesam A. Nasif, Mohammed H. Mukhtar, Hoda M. El-Emshaty, Ahmed H. Alwazna

**Affiliations:** 1 Department of Biochemistry, Faculty of Medicine, Umm Al-Qura University, Makkah, Kingdom of Saudi Arabia; 2 Molecular Biology Department, Genetic Engineering and Biotechnology Research Institute, Sadat City University, Sadat City, Egypt; 3 Research Laboratories, Gastroenterology Surgical Center, Faculty of Medicine, Mansoura University, Mansoura, Egypt; 4 Consultant Nephrologist, Nephrology Unit, Al-Rahma Hospital, Madinah, Saudi Arabia

**Keywords:** Human serum albumin, Vitamin D receptor activator, Hemodialysis patients, HPLC, Hyperparathyroidism, Oxidative albumin ratio

## Abstract

**Background:**

Hemodialysis (HD) patients with secondary Hyperparathyroidism (s-HPT) are exposed to increased inflammation and oxidative stress. In HD patients, oxidized albumin is a reliable marker of oxidative stress and its clinical significance has been rarely studied.

**Objective:**

The objective of this study was to evaluate Cys34 Human Serum Albumin (HSA) as oxidative stress biomarker in HD patients with s-HPT and its relationship with inflammation on bone turnover markers during oral calcitriol supplementation for vitamin D.

**Patients and Methods:**

Fifteen stable hemodialysis patients with s-HPT (mean age 48.67±8.15, 11 males and 4 females) were used in the experiment to receive calcitriol treatment for 16 weeks (0.25mcg or 0.5 mcg once a day according to serum level of Ca and P for each). The changes in the serum biochemical parameters (Ca, P, ALP, and iPTH), inflammatory markers (CRP and IL-6 levels) and serum oxidative stress condition (SOD, IS and albumin ratio HNA/HMA) were evaluated before and at 8 and 16 weeks of calcitriol treatment. The correlations between those factors were studied.

**Results:**

All patients responded to oral calcitriol therapy, with a significant decrease in the serum iPTH. The results showed that calcitriol could effectively suppress iPTH secretion with a significant elevation of serum Ca and P but ALP remained unchanged during the study. It can also effectively reduce the inflammatory markers (CRP and IL-6), while increasing the oxidative markers (SOD and IS). Oxidative albumin ratio HNA/HMA showed a significant (*p*=0.001) reduction after 16 weeks of calcitriol treatment and the redox state of HSA showed a positive prediction for hyperparathyroidism and for inflammation.

**Conclusion:**

The redox state of HSA could be used as a predictor for monitoring hyperparathyroidism and inflammation during calcitriol treatment by retarding albumin oxidation in HD patients with secondary hyperparathyroidism.

## 1 INTRODUCTION

Chronic renal failure is a progressive loss of renal function over a period of months or years. Hemodialysis is a suitable treatment that is usually given to the Chronic Renal Failure (CRF) patients who are not undergoing renal transplantation therapy [1]. In hemodialysis patients, the incidence of atherosclerotic diseases and cardiovascular risks are significantly high [2]. The nonconventional factors contribute to the increased risk of cardiovascular mortality in these patients, including oxidative stress, disorders of bone and mineral metabolisms in Chronic Kidney Disease (CKD-MBD), and chronic inflammation [3].

Reactive Oxygen Species (ROS), are highly reactive molecules, that produced by living organisms upon normal cellular metabolism and environmental factors. ROS can damage nucleic acids and proteins, thereby altering their functions. By producing antioxidants, the human body has many mechanisms to counteract oxidative stress [4]. Maintenance of this balance is crucial in biological systems, wherein cells are endowed with a molecular defense system, including antioxidants enzymes such as glutathione peroxidase, superoxide dismutase (SOD), catalase, and reduced glutathione (GOSH) as well as vitamins (A, C and E) [5]. In renal failure, the oxidant-antioxidant balance has been previously investigated. The dialysis treatment has been shown to increase oxidation markers in the blood of treated patients and lipid peroxidation in cell membranes [6], which lead to cardiovascular dysfunction.

Chronic Kidney Disease (CKD) results in a decrease in circulating active vitamin D levels, which has diverse impacts on metabolism and organ functions [7-9]. Secondary hyperparathyroidism (SHPT) is part of CKD-MBD and characterized by high amounts of parathyroid hormone (PTH) associated with high turnover bone disease and vascular calcification [10]. Treatment with vitamin D receptor activator (VDRA) ameliorates abnormalities in bone and mineral metabolism, cardiac function, and immune response [7-9]. However, the results suggested the potential benefits of both oral and intravenous VDRA in hemodialysis patients. While the precise mechanism for this effect is unknown, VDRA might have impacts beyond its known role in mineral metabolism [11].

The role of hyperparathyroidism in the genesis of inflammation/oxidative stress was suggested by the demonstration of the direct relationship between PTH levels and inflammatory markers in patients with primary hyperparathyroidism and in general population [12-14]. Oxidative stress has been incriminated in the development of pathological conditions, and direct evidence for *in vivo* oxidative stress in hemodialysis (HD) patients relied entirely on the determination of oxidation by-products of circulatory biomolecules [15].

**Fig. (1):**
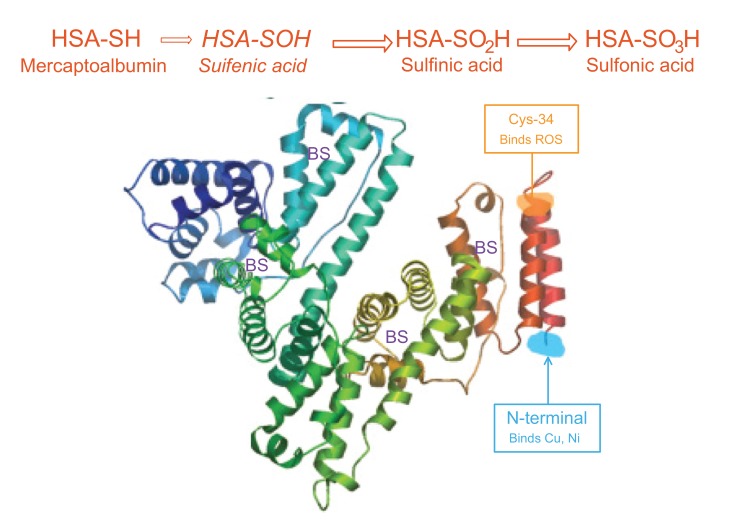
Albumin structure; Crystallographic studies stated that albumin HSA a heart-shaped tertiary structure with high α-helical content and 3 homologous domains, each comprised of 2 subdomains. The Cys34 (ORANGE) provides thiol properties conferring antioxidant ability to the protein. From the reduced form (mercaptoalbumin), albumin can be oxidized up to a highly oxidized state (sulfenic/sulfonic acid, known as nonmercaptoalbumin 2) The *N*-terminal (BLUE) acts as scavenge of free metals usually involved in pro-oxidant reactions, contributing to the antioxidant capacity of the molecule [13].

Oxidative stress might cause the reversible and/or irreversible modification of proteins [16]. In HD patients, HSA is the major plasma protein target of oxidative stress [17]. Depending on the redox state, separation of HSA gives three fractions according to cysteine-34 (Cys34): mercaptalbumin (HMA; reduced form) with a free thiol group on Cys-34, nonmercaptalbumin 1 (HNA1) with cysteine, homocysteine or glutathione bound by a disulfide bond and nonmercaptalbumin 2 (HNA2) with cysteine oxidized to sulfonic acid or sulfenic [18-20] (Fig. **1**). In healthy young persons, HNA1 accounted for 20-30%, HMA accounts for 70-80%, and HNA2 about 2-5% of total albumin. It has been reported that during several diseases and aging, the fractions of the oxidized forms were increased [21].

Since the redox status of a free thiol group in proteins could be an indicator for oxidative stress; measuring the redox status of Cys34 in HSA might allow the degree of organ damage by oxidative stress and the development of new antioxidative therapeutic approaches [22]. Thus, the aim of this study was to investigate the role of Cys34 HSA with other biochemical and inflammatory markers in evaluating HD patients with secondary hyperparathyroidism during calcitriol oral administration.

## 2 MATERIALS AND METHODS

This study was conducted on fifteen HD patients with sHPT (11 men and 4 women) aged from 26 to 62 years (mean age 48.67±8.15), undergone regular hemodialysis 3 times/week for at least 12 months before the study began. Informed consent was obtained from all patients, and the experimental protocol was approved by Ethics Review Board for Human Studies (Faculty of Medicine, Umm Al-Qura University, Makkah, KSA). All patients had no antioxidant (* i.e.*, vitamin E or C) supplement in the three months’ period before the dialysis. Patients were excluded when they received vitamin D therapy within 3 months from the study start.

A first blood sample was collected from each patient prior to initiating calcitriol treatment followed by 2^nd^ and 3^rd^ samples taken at a follow-up visit after 8 and 16 weeks of receiving oral calcitriol, respectively. The serum samples were immediately stored at -80°C until analysis. The calcitriol dosage calculated for each patient according to the level of Ca, P and iPTH. Eligible patients were those presenting with serum levels of Ca ≥8 mg/dl, P ≤5.5 mg/dl and iPTH ≥300 pg/mL.

### 2.1 Determination of Biochemical Markers

Serum levels of calcium were measured by the calcium-O-cresolphthalein complexone method [23] and phosphorus (P) by the phosphomolybdate method [24]. The analysis was performed on a Roche/Hitachi Modular P analyzer (CAN 210, Roche Diagnostics, Basal, Switzerland). Serum levels of calcium were adjusted for serum albumin wherein the lab reference ranges were 8.8-10.2 mg/dl for serum Ca and 2.5-5 mg/dl for serum P. Serum iPTH level was measured by IMMULITE and IMMULITE 1000 Analyzers for the quantitative measurement of iPTH (Siemens Healthcare Diagnostics, USA) [25]. The reference range for iPTH was 10-69 pg/mL, with analytical sensitivity 3 pg/mL and intra- and inter-assay coefficient of variation (CV) with values less than 5% and 7%, respectively. Serum alkaline phosphatase (ALP) was measured by colorimetric assay in accordance with a standardized method of the German Society of Clinical Chemistry [26]. Analytical sensitivity for the ALP assay was <1 IU/l (reference range between 98 IU/l and 279 IU/l) with an intra- and inter-assay variability lower than 10.1% and 10%, respectively.

### 2.2 Determination of Inflammatory Markers

C-Reactive Protein (CRP) assessed in the sera of HD patients using hs-CRP ELISA (Diagnostic Automation/Cortez Diagnostics, Inc., CA 91302 USA). The hs-CRP ELISA based on the principle of a solid phase ELISA [27]. The assay system utilizes mouse monoclonal anti-CRP antibody for solid-phase immobilization, on the microtiter wells, developed against a distinct antigenic determinant on the CRP molecule and conjugated HRP goat anti-CRP antibody. The test sample was allowed to react simultaneously with the 2 antibodies, which result in the CRP molecule being sandwiched between enzyme-linked antibodies and the solid phase. Tetramethyl Benzidine (TMB) reagent was added to develop the blue color and the reaction was stopped with 1N HCl changing the color to be yellow. The CRP concentration was determined spectrophotometrically at 450nm wherein the concentration was proportional to the color intensity.

### 2.3 Measurement of Interleukin-6 by ELISA

Serum IL-6 was quantitatively detected using human IL-6 ELISA kit (Boster Biological Technology, 3942B Valley Av, Pleasanton, CA, 94566), based on standard sandwich ELISA. Mouse monoclonal antibody specific for IL-6 was pre-coated on 96-well plates. Serum samples and standards (E.coli, p29-M212) were added to the wells with subsequent addition for a biotinylated polyclonal antibody specific for IL-6 (1:100 dilution) developed in goat, followed by washing with TBS or PBS buffer. The complex of Avidin-Biotin-peroxidase was added and after washing for excess unbound conjugate, HRP substrate TMB was added to visualize the enzymatic reaction as a blue product changed to yellow color after acidic-stop solution. The intensity of the yellow color is proportional to human IL-6 concentration and the OD absorbance was recorded at 450 nm in a microplate reader, within 30 min after adding the stop solution. The detection ranges were 4.69 pg/mL-300 pg/mL with sensitivity of <0.3 pg/mL.

### 2.4 Chromatographic Analysis of Human Serum Albumin

The redox status of HSA was analyzed using HPLC (high performance liquid chromatography) in HD patients as previously described [16] at 0, 8, and 16 weeks after oral calcitriol treatment. HSA is a mixture of nonmercaptalbumin (HNA; oxidized form) and mercaptalbumin (HMA; reduced form). HMA contains one highly reactive sulfhydryl group at position 34 (Cys34), while other serum proteins contain few or no high sulfhydryl groups. HNA is comprised of at least three types of molecules. The major HNA component is a mixed disulfide with glutathione (HNA-1) or cysteine. The other is a more highly oxidized product than the mixed disulfide, in which the thiol group is oxidized to the sulfinic (SO_2_H), sulfenic (SOH), and sulfonic (SO_3_H) states (HNA-2), the proportions of which are small in extracellular fluids [16]. In brief, HPLC analysis of serum aliquots (5µL) was performed using a Shodex Asohipak ES-502N column (Showa Denko Co., Tokyo, Japan). The column temperature was set at 35°C. A linear gradient elution (increasing ethanol concentrations from 0% to 5%) was prepared in a solution containing 0.05 mol/L sodium acetate and 0.4 mol/L sodium sulfate mixture at pH 4.85. The flow rate was 1.0 mL/min, from HPLC profiles of HSA. The values of HMA and HNA of HSA were estimated as the area of each fraction, divided by the total area of HSA peak. Oxidized albumin ratio (HNA/HMA) was determined and considered as a reliable marker for the extent of systematic oxidative damage [16, 28].

### 2.5 Measurement of the Circulating Indoxyl Sulfate (IS)

The circulating uremic toxin Indoxyl Sulfate (IS) was quantitatively detected using quantitative Sandwich ELISA kit (human Indoxyl sulfate ELISA kit, Cat. no. MBS019983). The serum sample, blank/control was added to a specific well, as designed with subsequent 50 µL of sample diluents, and 100 µL of HRP conjugated reagent was added to each well, then incubated at 37°C for 60 min. The chromogen solution was added, incubated in the dark for 15min and the developed color was changed from blue to yellow after the reaction was stopped. The Optical Density (OD) was read at 450 nm using MicroElisa Strip Plate reader, within 15 min after the addition of stop solution. The detection range of this kit is 3.12- 100 µg/mL, and the sensitivity is 1.0 µg/mL.

### 2.6 Analysis of Superoxide Dismutase (SOD)

Superoxide Dismutase (SOD) is a family of metalloproteins which catalyze the disputation of superoxide anion radicals (O_2_ˉ) to Hydrogen Peroxide (H_2_O_2_) and oxygen (O_2_). The level of SOD was measured by human Cu/Zn SOD platinum ELISA kit (www.eBioscience.com). Antihuman Cu/Zn SOD antibody coating to microwells was used to capture human Cu/Zn SOD present in sera of HD patients, then, HRP-conjugated anti-human Cu/Zn SOD antibody was added, followed by the substrate. The reaction was terminated by acid and the absorbance was measured at 450nm. The colored product was formed in proportion to the level of human Cu/Zn SOD found in the sample.

### 2.7 Statistical Analysis

Statistical analysis was carried out using SPSS 17.0 (version 17, Sydney, NSW, Australia). Quantitative data was expressed as mean ±SD, depending on the nature of the data. Comparison between means was tested by Wilcoxon Signed Rank test and the significance was considered at *p*<0.05. Relationship (*r*) between variables was detected by Pearson's correlation coefficient and logistic regression analysis was carried out to analyze the prediction.

## 3 RESULTS

This investigation included 15 patients (11 male and 4 females) on hemodialysis with age ranging from 26 to 62 years (mean 48.67±8.15). Sera were collected from all patients before calcitriol administration (0.25 mcg or 0.5 mcg once a day according to the level of serum Ca and P for each patient) and during therapy (at 8 and 16 weeks of treatment).

### 3.1 Determination of Biochemical and Inflammatory Markers

All samples were investigated for biochemical and inflammatory markers before and after calcitriol therapy according to the protocol and the data was recorded in Table **1**. Serum level of Ca and P showed a significant elevation at both 8 weeks (8.086±0.488 mg/dl, **p*=*0.048 and 3.85±0.32 mg/dl, *p*=0.001) and 16 weeks (8.4±0.6 mg/dl, **p*=*0.001 and 4.1±0.366 mg/dl, *p*=0.001) of calcitriol therapy compared with that recorded before therapy (7.93±0.576 mg/dl, 3.64+0.35 mg/dl, respectively) whereas the ALP (IU/L) remained unchanged through the time course of the study. Serum level of iPTH pretreatment (403.2±76.98 pg/ml) was reduced significantly (**p*=*0.001) after 8 weeks and 16 weeks of calcitriol oral therapy (373.7±79.64 pg/ml, 338.6±77.45 pg/ml) respectively.

Serum level of CRP and inflammatory cytokine IL-6 were reduced significantly at 8 weeks (20.46±6.7mg/L, *P*=0.0l and 25.34±10.279pg/ml, *P*=0.027 respectively) and 16 weeks (16.11±7.23mg/L, *P*=0.001 and 22.47±10.95pg/mL, *P*=0.005 respectively) of oral calcitriol therapy compared to that recorded pre-treatment (23.17±8.11mg/L and 27.45±11.97pg/ml respectively). Serum level of CRP and IL-6 were reduced by 11.7% and 7.68% at 8 weeks and 30.5% and 18.14% at 16 weeks of treatment respectively.

### 3.2 Assessment of Oxidative Stress Biomarkers in Serum

Table **2** presents the serum amounts of oxidative stress biomarkers in HD patients during the study. Serum Indoxyl Sulfate (IS) showed a significant elevation in patients on hemodialysis only at 16 weeks (79.0±13.73 µg/mL* vs *74.066±15.65 µg/mL, *P*=0.01) post-treatment compared with that pre treatment. While serum anti-oxidant SOD after calcitriol administration was highly elevated (*p*=0.001) after both 8 weeks (623.9±156.9 ng/mL) and 16 weeks (601.9±160.1 ng/mL) post-treatment compared with the level pretreatment (387.87±106.3 ng/mL) of calcitriol.

The oxidative status of serum albumin in HD patients was measured by HPLC pre and post oral administration of calcitriol therapy. The un-oxidized form (HMA) and oxidized form (HNA) of HSA were calculated as the area of each fraction divided by the total area of HSA peak. As listed in Table **2**, the percent of oxidized albumin (HNA%) was significantly (*p*=0.001) decreased at both 8 weeks (48.36±4.56) and 16 weeks (52.9±6.03) post-therapy compared with the level pre treatment (62.356±4.3). The percent of reduced albumin (HMA%) before therapy (37.5±4.38) was significantly (*p*=0.001) increased at both 8 weeks (51.64±4.56) and 16 weeks (46.4±4.64) post calcitriol oral therapy. However, oxidative albumin ratio HNA/HMA showed significant (*p*=0.001) reduction only at 16 weeks (1.15±0.21) of calcitriol oral therapy compared to that pre-treatment (1.86±0.3).

### 3.3 Correlation Between Biochemical, Oxidative and Inflammatory Markers with iPTH Level

**Fig. (2):**
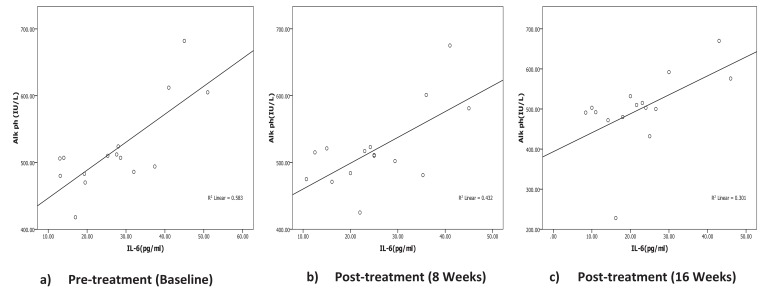
Correlation between ALP and IL-6 pre and post treatment.

Correlation between biochemical, oxidative and inflammatory markers with iPTH Level was listed in Table **3**. Serum iPTH showed significant negative correlation (r=-0.559, *p*=0.031) with Ca only pre-treatment but after calcitriol oral therapy, no significant correlation was recorded between iPTH with Ca, P or ALP at any time during the study period. Correlation analysis of iPTH with inflammatory markers (IL-6 and CRP) showed no statistically significant correlation pre- or post-treatment. Serum ALP showed significant positive correlation with IL-6 (Fig. **2**, Table **3**) pretreatment (r=0.763, *p*=0.001) and after treatment (r=0.657, *p*=0.008) (r=0.549, *p*=0.034) at 8 and 16 weeks post-therapy. There is no association between ALP with any other inflammatory or oxidative biomarkers through the study period.

**Fig. (3):**
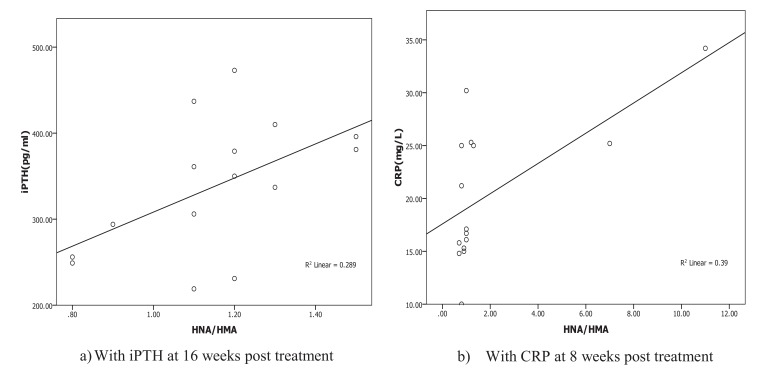
Correlation between HNA/HMA ratio with iPTH at 16 weeks and with CRP at 8 weeks post treatment.

**Fig. (4):**
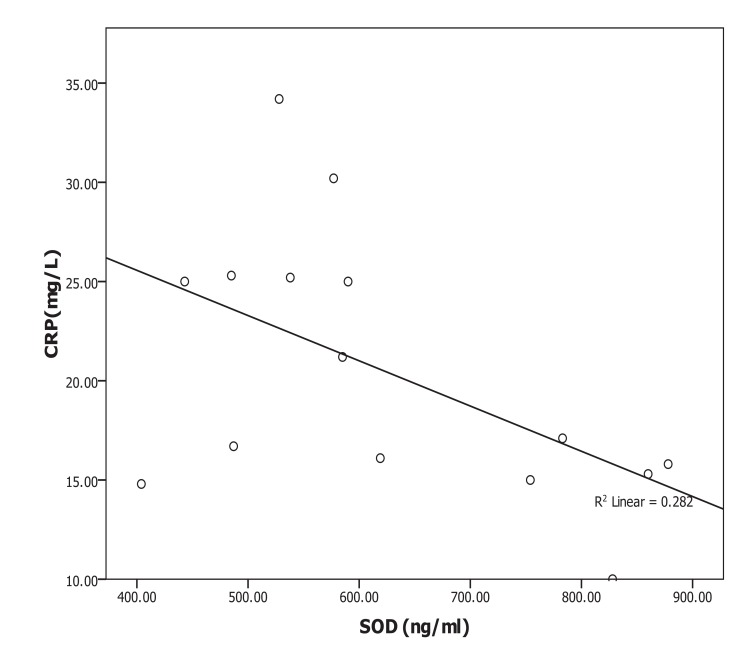
Correlation between CRP with SOD at 8 Weeks Post-treatment.

Oxidative albumin ratio HNA/HMA was associated with iPTH at 16 weeks of calcitriol oral therapy (r=0.537, *p*=0.039) suggesting that, HNA/HMA could be used as a marker for evaluating the reduction in hyperparathyroidism during calcitriol oral therapy. HNA/HMA was also associated with inflammatory marker CRP (r=0.625, *p*=0.013) at 8 weeks of treatment (Fig. **3**) but negative association (r=-0.531, *p*=0.04) was recorded between CRP with SOD at 8 weeks of treatment (Fig. **4**). These results could be explained as the reduction in oxidative albumin ratio HNA/HMA may be through an elevation in anti-oxidant SOD and reduction in inflammation. However, the oxidized albumin HNA% showed a negative association with inflammatory marker IL-6 at 16 weeks post-treatment (r=-0.638, *p*=0.011) suggesting that the ratio of oxidized albumin could reflect the degree of immunomodulatory action as measured by cytokine IL-6 during treatment.

**Fig. (5):**
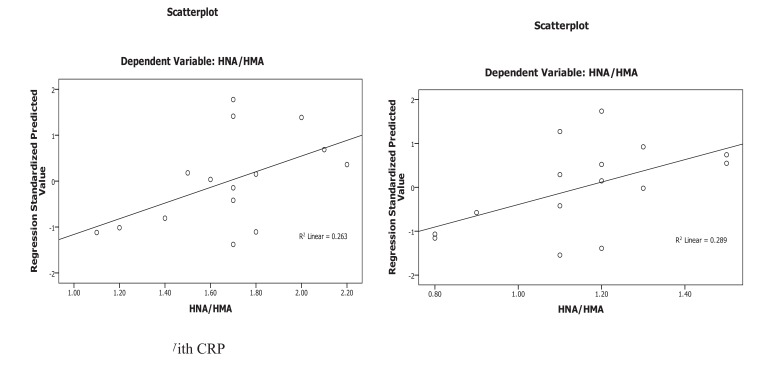
Scatter plot for HNA/HMA Ratio with CRP and iPTH.

Linear regression analysis of oxidized albumin ratios with inflammation and iPTH showed that the HNA/HMA was detected as a predictor for inflammation measured by CRP (B=0.273, *p*=0.013) at 8 weeks of treatment and significant prediction was also recorded by linear regression (B=0.001, *p*=0.039) with iPTH at 16 weeks post oral calcitriol therapy indicating that the redox state of HSA could be used for prediction of inflammation and hyperparathyroidism in HD patients during therapy (Fig. **5**).

## 4 DISCUSSION

Oxidative stress and inflammation recently came into focus as non-conventional risk factors of cardiovascular morbidity and overall mortality in the end stage of renal disease [1]. There is a great interest to develop diagnostic tools to control the extent of oxidative damage in organs or tissues and the use of bioactive antioxidants in the prevention or treatment of oxidative stress-related diseases [29, 30]. Therefore, the goal of this investigation was to study Cys34 HSA as oxidative stress biomarker in HD patients with sHPT and its relationship with various biochemical and inflammatory markers during VDRA calcitriol oral therapy.

Therapy with vitamin D for hemodialysis patients was initially designed to test sHPT, phosphorus absorption and regulating calcium in the intestine and bone remolding [31]. Intact parathyroid hormone (iPTH), phosphorus and calcium levels were determined for all patients on hemodialysis before and after VDR calcitriol therapy in our experiment. We noted that the high levels of iPTH were significantly reduced after 8 weeks of treatment, meanwhile the base line levels of calcium and phosphorus were increased significantly for Ca (*p*=0.048) and P (*p*=0.001). On contrary, *Tanaka * et al*.* [11] in their study for 4 weeks on HD patients with sHPT treated with calcitriol at an intravenous dose of 1.5µg/week reported that, there were no significant changes in serum iPTH, Ca or P levels after treatment. They concluded that these results are not surprising because they applied low doses of VDRA in their study wherein sHPT is resistant to vitamin D therapy, partly due to the decreased parathyroid vitamin D receptors. However, *Izquierodo * et al*.* [31] in their study on HD patients showed that high base line iPTH level was decreased significantly after treatment with paricalcitol for 12 weeks while both blood phosphorus and calcium levels were increased.

*Wu * et al*.* [32] reported in a study on high levels of PTH with 25 hemodialysis patients for 16 weeks compared with 20 controls that the treatment with calcitriol reduced not only the levels of PTH, but also decreased inflammatory (CD cytokines, CRP and IL-6) and oxidative stress markers (TRAP). In the current study, reduction in PTH and inflammatory markers (CRP, IL-6) was observed at 8 weeks of oral calcitriol therapy; nevertheless, there is no significant correlation was recorded between them at 8 weeks or even at 16 weeks post-treatment. Therefore, the reduction in inflammation in HD patients after calcitriol oral administration is unlikely to be due to improvement in PTH level. However, the results of another descriptive study suggested that the oxidative stress and inflammation of HD patients might have arisen from mechanisms other than SHPT [33].

Lower amounts of serum vitamin D might be associated with both elevated serum alkaline phosphatase and inflammatory markers [34, 35]. Therefore, serum alkaline phosphatase in CKD might reflect atherogenic milieu and an inflammatory but not merely a biomarker of bone mineral metabolism. In the current study, serum ALP was elevated in hemodialysis patients with no significant changes through the time course of treatment and a significant association was recorded between ALP and IL-6 pre-treatment (r=0.763, *p*=0.001) and during therapy (at 8 weeks r=0.657, *p*=0.008 and at 16 weeks r=0.549, *p*=0.03). These could be an explanation for the association of serum ALP with the mortality in CKD population is inflammation [36].

Modulatory influence on *in vitro* inflammatory and immune signaling pathways have been described, with a high reduction of proinflammatory cytokines IL-8, CRP and TNF-α as well as an increase in the anti-inflammatory markers [37, 38]. Consistent with these studies, our results showed significant reduction in inflammatory markers CRP and IL-6 in hemodialysis patients after oral calcitriol treatment and the reduction was observed with CRP by 11.7% at 8 weeks and 30.47% reduction at 16 weeks and for IL-6, the reduction was 7.68% at 8 weeks and 18.14% at 16 weeks post-treatment. *Izquierdo * et al*.* [31] and *Alborazi * et al*.* [39] recorded 20% reduction in CRP with paricalcitol.

The utility of detecting the redox status of Cys34 in HSA as a marker for oxidative stress in the circulation was studied by evaluating the degree of oxidized Cys34 HSA using HPLC [22]. In a healthy young person, HMA accounts for 70-80%, HNA1 accounts for 20-30% and HNA2 accounts for 2-5% of total albumin. It has been described that during numerous diseases and aging, the fractions of the oxidized forms are increased [21]. In the current study, a significant decrease (*p*=0.001) of HNA and significant increase of HMA (*p*=0.001) was recorded at 8 weeks post calcitriol therapy but the oxidized albumin ratio HNA/HMA was significantly (*p*=0.001) reduced only after 16 weeks post-treatment. However, *Tanaka * et al*.* [11] reported that, among the oxidative stress biomarkers in their study, only the oxidized albumin ratio was significantly affected and changed by intravenous calcitriol treatment.

Oxidative stress occurring due to decreased anti-oxidant defenses and an increase in pro-oxidant factors is a well-recognized phenomenon in hemodialysis patients [1]. *Tanaka * et al**. [11] concluded that, indoxyl sulfate, a representative uremic toxin known to form Reactive Oxygen Species (ROS) and SOD activities in serum remained unchanged during calcitriol therapy study period on HD patients with sHPT [28, 40]. Our results showed that SOD was elevated significantly (*p*=0.001) during the time course of treatment but serum IS was not changed during therapy. It is well known that some uremic toxins including indoxyl sulfate could produce ROS [40] and increase the oxidized albumin ratio [28]. However, neither the indoxyl sulfate nor SOD has any significant association with the oxidized albumin ratio. Therefore, the oral calcitriol therapy-induced changes in oxidized albumin ratio or improves the oxidative status in HD patients by a mechanism other than the reduction in indoxyl sulfate or elevation in SOD activity. However, SOD in the current study showed negative association (r=-0.531, *p*=0.04) with inflammatory marker CRP only at 8 weeks of calcitriol oral therapy. Therefore, SOD scavenging activity for oxidative stress could be through the improvement of the inflammation.

Patients at the end-stage renal disease have higher levels of oxidative stress and inflammation than the normal population. Several factors contributed to these issues, wherein the parathyroid hormone is also implicated [33]. Consistent with these studies, higher levels of inflammatory markers (* i.e.*, CRP, IL-6), oxidative stress and hyperparathyroidism were recorded in HD patients. The iPTH was correlated significantly with oxidative albumin ratio HNA/HMA at 16 weeks (1.68±0.3* vs *1.15±0.21, **p*=0.001*) post-treatment. Linear regression for HNA/HMA showed the significant prediction for iPTH at 16 weeks post oral calcitriol therapy (B=0.001, *p*=0.039) and for inflammation as measured by CRP (B=0.273, *p*=0.013) at 8 weeks post calcitriol oral therapy. Therefore, the improvement of oxidative stress as measured by HNA/HMA is likely to be through amelioration of iPTH by oral administration of calcitriol therapy at 16 weeks of treatment and may be mediated through improvement in inflammation.

However, the mechanisms through which VDRA decrease the oxidative stress are not clearly understood. There is no evidence showing that VDRA exhibits antiradical activities [11] wherein one possible explanation for the impact of VDRA is that VDRA resulted in the modulation of the immune and inflammatory systems, and thereby improve the oxidative stress [41]. In current study, significant reduction in inflammatory markers (IL-6, CRP) at 8 weeks of oral calcitriol therapy and significant correlation was recorded between CRP and HNA/HMMA (r=0.625, *p*=0.013) at 8 weeks of calcitriol treatment assuming that the changes in albumin redox status may be due to the anti-inflammatory action of calcitriol oral therapy after 8 weeks of treatment. Therefore, the treatment with calcitriol, a non-selective vitamin D activator, decreased the inflammatory biomarkers [34] and had immunomodulatory impacts [11] therefore, showing the effect to reduce cardiovascular mortality and damage, beyond the intended control of sHPT [42].

## CONCLUSION

We have applied HPLC techniques to measure the oxidized albumin ratio and to demonstrate that albumin is the predominant oxidatively modified plasma protein in HD patients with secondary hyperparathyroidism. Oxidation of albumin could decrease the plasma antioxidant defenses and increase the likelihood of oxidant stress. Oral calcitriol supplementation; corrects vitamin D deficiency, retarding the albumin oxidation, decreases inflammation and suppresses iPTH secretion in a short follow up period. Our study highlights the redox state of HSA might be used as a predictor for evaluating hyperparathyroidism, oxidative status and inflammation in HD patients with secondary hyperparathyroidism.

## Figures and Tables

**Table 1:** Serum Levels of Biochemical and Inflammatory Markers for Calcitriol Therapy in Patients on HD with s-HPT.

**Serum** **Marker** (Unit)	**HD Patients with Calcitriol Therapy** Mean ±SD (95% CI))			***p* Value**
	**Pre-treatment** (Baseline)	**Post-treatment**		
		**8 Weeks**	**16 Weeks**	
**Ca** (mg/dl)	7.93 ±0.576 (7.61 - 8.253)	8.086 ±0.488 * (7.816 - 8.357)	8.4 ±0.6^** ##^ (8.065 - 8.73)	* 0.048 ** 0.001 ^##^ 0.003
**P** (mg/dl)	3.64 ±0.35 (3.45 - 3.84)	3.85 ±0.32 ** (3.67 - 4.03)	4.1 ±0.366 ^** ##^ (3.89 - 4.3)	** 0.001 ** 0.001 ^##^ 0.003
**ALP** (IU/L)	519.7 ±65.65 (483.37 - 556.09)	519.46 ±60.43 (486.0 - 552.9)	449.7 ±94.29 (477.5 - 551.94)	>0.05
**iPTH** (pg/ml)	403.2 ±76.98 (360.56 - 445.8)	373.7 ±79.64 ** (329.6 - 417.8)	338.6 ±77.45 ^** ##^ (295.7 - 381.49)	** 0.001 ** 0.001 ^##^ 0.001
**CRP** (mg/L)	23.17 ±8.11 (18.68 - 27.66)	20.46 ±6.7 * (16.729 - 24.19)	16.11 ±7.23 ^** ##^ (12.105 - 20.13)	* 0.01 ** 0.001 ^##^ 0.001
**IL-6** (pg/ml)	27.45 ±11.97 (20.8 - 34.08)	25.34 ±10.279 * (19.65 - 31.04)	22.47 ±10.95 ^**#^ (16.4 - 28.53)	* 0.027 ** 0.005 ^#^ 0.011

*Data* *: Expressed as mean ±SD (95%CI of mean)* *Markers* *:* *Ca* *: Calcium,* *P* *: phosphorus,* *ALP* *: Alkaline Phosphatase,* *iPTH* *: Intact Parathyroid Hormone,* *CRP* *: C Reactive Protein,* *IL-6* *: Interleukin 6* *The comparison between means was assessed using Wilcoxon Signed Rank test at 16 weeks post treatment was considered significant at* *p Value* *:* * *<0.05 &* ** *<0.005 compared to pre-treatment and* ^#^ *<0.05 &* ^##^ *<0.005 compared to 8 weeks post-treatment.* Serum level of CRP was reduced by 11.7% at 8 weeks and 30.5% at 16 weeks of treatment Serum level of IL-6 was reduced by 7.68% at 8 weeks and 18.14% at 16 weeks of treatment

**Table 2:** Serum Oxidative Stress Markers and HSA Status in HD Patients with sHPT.

**Serum Marker** (Unit)	**HD Patients with Calcitriol Therapy** Mean ±SD (95% CI)			***p* Value**
	**Pre-treatment** (Baseline)	**Post-treatment**		
		**8 Weeks**	**16 Weeks**	
**IS** (µg/ml)	74.066±15.65 (65.4 - 82.7)	77.13±14.9 (68.85 - 85.41)	79.0±13.73 * (71.39 - 86.6)	* 0.01
**SOD** (ng/ml)	387.87±106.3 (329.0 - 446.7)	623.9±156.9 ** (537.0 - 710.8)	601.9±160.1 ** (513.25 - 690.6)	** 0.001
**HNA** (%)	62.356±4.3 (59.96 - 64.7)	48.36±4.56 ** (45.83 - 50.88)	52.9±6.03 ^** ##^ (49.57 - 56.26)	** 0.001 ^##^ 0.002
**HMA** (%)	37.51±4.38 (35.08 - 39.93)	51.64±4.56 ** (49.1 - 54.17)	46.4±4.64 ^** ##^ (43.8 - 48.98)	** 0.001 ^##^ 0.001
**HNA/HMA** (ratio)	1.68±0.3 (1.514 - 1.85)	2.006±2.94 (0.376 - 3.64)	1.15±0.21 ** (1.04 - 1.27)	** 0.001

*Data* *: Expressed as mean ±SD (95%CI of mean)* *Markers* *:* *SOD* *: Superoxide dismutase,* *IS* *: Indoxyl Sulfate,* *HNA* *: Human non-mercaptoalbumin,* *HMA* *: Human mercaptoalbumin,* *HNA/HMA* *: Oxidized albumin ratio* The comparison between means was assessed using Wilcoxon Signed Rank test at 16 weeks post treatment was considered significant at * p Value:* * *<0.05 &* ** *<0.005 compared to pre-treatment and* ^##^ *<0.005 compared to 8 weeks post-treatment.*

**Table 3:** Pearson correlation between oxidative albumin with inflammatory and biochemical markers in HD Patients Pre- and post- Calcitriol Therapy.

**Correlations**	**Pre-Therapy**		**Post- Therapy**			
	r	*p*	**After 8 weeks**		**After 16 weeks**	
			r	*p*	r	*p*
**IL-6 with ALP**	0.763	0.001	0.657	0.008	0.549	0.034
**HNA with HMA**	0.997	<0.0001	-0.100	<0.0001	-0.916	<0.0001
**HNA/HMA with HNA**	0.991	<0.0001	0.103	0.7	0.904	<0.0001
**HNA/HMA with HMA**	-0.993	<0.0001	-0.103	0.7	-0.99	<0.0001
**HNA/HMA with iPTH**	0.513	0.05	0.127	0.065	0.537	0.039
**HNA/HMA with CRP**	0.321	0.24	0.625	0.013	0.387	0.154
**CRP with SOD**	-0.133	0.637	-0.531	0.042	0.414	0.125
**iPTH with Ca**	-0.558	0.031	0.305	0.268	0.298	0.281
**iPTH with HMA**	-0.539	0.038	-0.257	0.354	-0.547	0.035

*Data: expressed as an association between variable (r); (negative (–) correlation)* *Ca: Calcium, P: Phosphate, ALP: Alkaline Phosphatase, iPTH: Intact Parathyroid Hormone, CRP: C Reactive Protein, IL-6: Interleukin 6, SOD: Superoxide dismutase, IS: Indoxyl Sulfate, HNA: Human non-mercaptoalbumin, HMA: Human mercaptoalbumin, HNA/HMA: Oxidized albumin ratio* *The correlation between variables was assessed using Person Correlation Coefficient and was considered significant at p Value:* * *<0.05 &* ** *<0.005 compared to other variable*
